# Supplementary material for: Predation and fragmentation portrayed in the statistical structure of prey time series
Source: BMC Ecol. 2009 May 6;9:10. doi: 10.1186/1472-6785-9-10 (PMC2689204; doi:10.1186/1472-6785-9-10)
Supplement: Additional file 2 — Voles and related classes ODDox Documentation. ODDox documentation of the agent-based model (ALMaSS) applied by Hendrichsen et al. The documentation is started by activating main.html. [file 1472-6785-9-10-S2.zip › Vole_ODDox/globals_0x63.html]

ALMaSS ODDox: Class Members

- Main Page
- Related Pages
- Classes
- Files

- File List
- File Members

- All
- Functions
- Variables
- Typedefs
- Enumerations
- Enumerator
- Defines

- \_
- a
- b
- c
- d
- f
- g
- h
- i
- l
- m
- n
- o
- p
- r
- s
- t
- u
- v
- w

Here is a list of all file members with links to the files they belong to:

### - c -

- cattle\_out
  : treatment.h- cattle\_out\_low
    : treatment.h- cfg\_CatastropheEventStartYear()
      : PopulationManager.cpp- cfg\_CIPEGridOutput\_day()
        : PopulationManager.cpp- cfg\_CIPEGridOutput\_day\_b()
          : PopulationManager.cpp- cfg\_CIPEGridOutput\_filename()
            : PopulationManager.cpp- cfg\_CIPEGridOutput\_filenameB()
              : PopulationManager.cpp- cfg\_CIPEGridOutput\_Interval()
                : PopulationManager.cpp- cfg\_CIPEGridOutput\_used
                  : VolePopulationManager.cpp
                  , Vole\_all.cpp
                  , PopulationManager.cpp- cfg\_DayInMonth()
                    : PopulationManager.cpp- cfg\_extradispmort
                      : VolePopulationManager.cpp
                      , Vole\_all.cpp- cfg\_f1sterilitychance()
                        : VolePopulationManager.cpp- cfg\_fixed\_random\_sequence()
                          : PopulationManager.cpp- cfg\_fungi\_app\_prop
                            : WinterWheat.cpp- cfg\_geneticproductfertilityeffect()
                              : VolePopulationManager.cpp- cfg\_geneticsterilitychance()
                                : VolePopulationManager.cpp- cfg\_GrassStartGrowth()
                                  : VolePopulationManager.cpp- cfg\_greg\_app\_prop
                                    : WinterWheat.cpp- cfg\_herbi\_app\_prop
                                      : WinterWheat.cpp- cfg\_ins\_app\_prop
                                        : WinterWheat.cpp- cfg\_MoveToLessFavourable()
                                          : VolePopulationManager.cpp- cfg\_organic\_extensive()
                                            : farm.cpp- cfg\_owl\_breed\_day()
                                              : Predators.cpp- cfg\_owl\_breed\_threshold()
                                                : Predators.cpp- cfg\_owl\_death\_threshold()
                                                  : Predators.cpp- cfg\_owl\_StartingNo()
                                                    : Predators.cpp- cfg\_PesticideAccumulationThreshold()
                                                      : Vole\_all.cpp- cfg\_pm\_eventfrequency()
                                                        : PopulationManager.cpp
                                                        , VolePopulationManager.cpp- cfg\_pm\_eventsize
                                                          : VolePopulationManager.cpp
                                                          , PopulationManager.cpp- cfg\_productapplicendyear
                                                            : Vole\_all.cpp- cfg\_productapplicstartyear
                                                              : Vole\_all.cpp- cfg\_ReallyBigOutput\_filename()
                                                                : PopulationManager.cpp- cfg\_ReallyBigOutput\_used()
                                                                  : PopulationManager.cpp
                                                                  , VolePopulationManager.cpp- cfg\_ReallyBigOutputMonthly\_used
                                                                    : VolePopulationManager.cpp
                                                                    , Vole\_all.cpp- cfg\_RipleysOutput\_day()
                                                                      : PopulationManager.cpp- cfg\_RipleysOutput\_filename()
                                                                        : PopulationManager.cpp- cfg\_RipleysOutput\_interval()
                                                                          : PopulationManager.cpp- cfg\_RipleysOutput\_used()
                                                                            : PopulationManager.cpp
                                                                            , VolePopulationManager.cpp
                                                                            , Vole\_all.cpp- cfg\_RipleysOutputFirstYear()
                                                                              : PopulationManager.cpp- cfg\_RipleysOutputMonthly\_used()
                                                                                : PopulationManager.cpp- cfg\_setaside\_glyphosate()
                                                                                  : setaside.cpp- cfg\_UseEasyPop()
                                                                                    : PopulationManager.cpp- cfg\_vole\_max\_age()
                                                                                      : Vole\_all.cpp- cfg\_vole\_starting\_number()
                                                                                        : VolePopulationManager.cpp- cfg\_VoleCatastrophe\_interval()
                                                                                          : PopulationManager.cpp- cfg\_VoleCatastrophe\_mortality()
                                                                                            : PopulationManager.cpp- cfg\_VoleCatastrophe\_on()
                                                                                              : PopulationManager.cpp- cfg\_weasel\_breed\_day()
                                                                                                : Predators.cpp- cfg\_weasel\_breed\_threshold()
                                                                                                  : Predators.cpp- cfg\_weasel\_death\_threshold()
                                                                                                    : Predators.cpp- cfg\_weasel\_DispersalMax()
                                                                                                      : Predators.cpp- cfg\_weasel\_home\_range()
                                                                                                        : Predators.cpp- cfg\_weasel\_kill\_efficiency()
                                                                                                          : Predators.cpp- cfg\_weasel\_NoFailuresBeforeDispersal()
                                                                                                            : Predators.cpp- cfg\_weasel\_search\_area()
                                                                                                              : Predators.cpp- cfg\_weasel\_StartingNo()
                                                                                                                : Predators.cpp- cut\_to\_hay
                                                                                                                  : treatment.h- cut\_to\_silage
                                                                                                                    : treatment.h- cut\_weeds
                                                                                                                      : treatment.h

---

Generated on Thu Jan 22 14:13:48 2009 for ALMaSS ODDox by 
 1.5.6
